# Perceived Sexual Orientation Based on Vocal and Facial Stimuli Is Linked to Self-Rated Sexual Orientation in Czech Men

**DOI:** 10.1371/journal.pone.0082417

**Published:** 2013-12-16

**Authors:** Jaroslava Varella Valentova, Jan Havlíček

**Affiliations:** 1 Center for Theoretical Study, Charles University and the Academy of Sciences of the Czech Republic, Prague, Czech Republic; 2 Department of Zoology, Faculty of Science, Charles University, Prague, Czech Republic; University of Goettingen, Germany

## Abstract

Previous research has shown that lay people can accurately assess male sexual orientation based on limited information, such as face, voice, or behavioral display. Gender-atypical traits are thought to serve as cues to sexual orientation. We investigated the presumed mechanisms of sexual orientation attribution using a standardized set of facial and vocal stimuli of Czech men. Both types of stimuli were rated for sexual orientation and masculinity-femininity by non-student heterosexual women and homosexual men. Our data showed that by evaluating vocal stimuli both women and homosexual men can judge sexual orientation of the target men in agreement with their self-reported sexual orientation. Nevertheless, only homosexual men accurately attributed sexual orientation of the two groups from facial images. Interestingly, facial images of homosexual targets were rated as more masculine than heterosexual targets. This indicates that attributions of sexual orientation are affected by stereotyped association between femininity and male homosexuality; however, reliance on such cues can lead to frequent misjudgments as was the case with the female raters. Although our study is based on a community sample recruited in a non-English speaking country, the results are generally consistent with the previous research and thus corroborate the validity of sexual orientation attributions.

## Introduction

Within European and American cultures, there is a widespread belief that men’s sexual orientation can be accurately judged solely on the basis of limited information, such as appearance, voice, or nonverbal behavior [1]. The ability to detect one’s sexual orientation is commonly called ‘gaydar’ and a high percentage of gay men believe their gaydar is effective [2]. The ability to detect like-oriented individuals may provide help, for instance, in the context of seeking romantic partners or other in-group members in general. A body of research (reviewed by Tskhay and Rule [3]) has shown that not only sexual orientation, but also other perceptually ambiguous characteristics, such as political orientation, can be identified above a chance level, although they do not show any obvious markers of belonging to the given social group. This supposedly automatic categorization of unknown individuals based on the first impression appears to be one of the most important mechanisms allowing people to quickly and effectively classify the social world.

Thus, sexual orientation is supposed to be a perceptually ambiguous characteristic, and during the last two decades more than twenty studies have been conducted on this topic. Previous work has, for example, tested the influence of behavioral characteristics, including voice, on sexual orientation attribution. It was found that independent listeners could distinguish between the speech of homosexual and heterosexual men [4]–[6], and observers were also able to accurately judge sexual orientation from short video-recordings [7]–[9]. Other research reported that sexual orientation can be estimated from still photographs of the entire clothed figure [6], [7], [10].

Further, recent studies showed relatively accurate judgments of sexual orientation based on facial photos. Moreover, accurate judgments were made as quickly as in 50 ms and the accuracy did not improve with the length of exposure [11]. This indicates that even a very short exposure to thin slices of information is enough to assess sexual orientation of unknown individuals. Furthermore, even isolated facial traits, such as hair, mouth, and eye area, provided sufficient information to enable above-chance accuracy in attributing sexual orientation [12]. Accuracy of sexual orientation attribution was higher than chance not only for upright facial pictures, but also for pictures presented upside-down, although under this condition, the accuracy was significantly reduced [13]. Recently, it has been reported that salience of behavioral and facial displays for sexual orientation judgments shows acceptable cross-cultural agreement [14], [15].

Several lines of empirical evidence suggest that, on average, homosexual individuals display personality and behavior traits typical of the opposite gender [1], [16], [17]. In line with this assumption, gender-atypical traits have been considered as cues to sexual orientation. Indeed, feminine male faces received higher ratings of homosexuality than masculine faces, although actual sexual orientation was not assessed [18]. This stereotype also affects judgments of individuals from gender-biased populations, so that it was shown that Asian men and Black women, rather than the opposite, were more frequently rated as homosexual [19]. Once heterosexual and homosexual individuals were compared, voices and behavior of homosexual males tended to be perceived as more feminine than those of their heterosexual counterparts [8], [9], [20].

Compared to heterosexuals, homosexual men show specific vocal patterns, some of them being rather female-like. For instance, homosexual males produce an expanded space in some vowels (here, the vowel/l/) more than heterosexual men, which is similar, although not identical, to heterosexual women [21]. On the other hand, despite the stereotype that homosexual men have female-like vocal traits and higher voice pitch in particular (even actors playing a gay role use higher-pitched voices [22]), the empirical data do not show any differences in mean voice pitch between homosexual and heterosexual men [4], [23]. Further, other research showed differences between homosexual and heterosexual men in vocal traits that usually do not differ between the sexes. For example, homosexual men produce higher negative skew in the fricative/s/[24], and they show higher frequency of lisping than heterosexual men, and women [25].

### 1.1 The Current Study

Our aim was to broaden the current state of knowledge about attributions of sexual orientation based on vocal and facial stimuli and the role of perceived masculinity-femininity in such attributions. Previous studies on this topic have mostly been done on the US population, with target stimuli downloaded from internet websites, and with undergraduate students as raters. Following the arguments proposed by Henrich et al. [26], that cultural practices may have crucial impact on psychological functioning and that generalization of findings based on student samples might frequently be limited, we primarily aimed to replicate the previous studies on 'gaydar' while employing distinct methods, such as recruitment of a community-based sample. As various communities may hold different stereotypes about the appearance of homosexual individuals, the studies based on samples taken from different cultures are of relevant value as they contribute to our understanding about the generality of the studied phenomena (i.e. here sexual orientation attribution). Further, the target sample was recruited in a Czech homosexual community which for many socio-historical reasons might differ from an American gay community.

Previous studies investigating vocal stimuli were performed on English-speaking participants. However, speech in individual languages varies considerably in terms of pitch variation, tonality, use of fricative/s/, and numerous others aspects which may affect sexual orientation judgments. Thus, one of the goals of the current study was to test whether ratings of sexual orientation based on vocal recordings in another language, in this case Czech (Slavic family of languages) correlate with self-reported sexual orientation of the rated men. It has been previously proposed that vocal displays might contain cues to sexual orientation and that such cues may function across languages [27]. So far, a binational study presented video recordings (which contained speech) of heterosexual and homosexual men, and it was shown that gaydar could work cross culturally [14]. We thus hypothesized that in line with previous research, perceived sexual orientation based on short vocal recording in the Czech language would be in agreement with self-reported sexual orientation of the rated men. We also examined voice pitch in relation to self-reported and judged sexual orientation and masculinity-femininity.

With respect to faces, in this study we have focused on facial morphology, minimizing social cues. Facial traits may not be as informative of sexual orientation when facial photograph stimuli are standardized and, thus, cues such as facial jewelry or involvement of facial muscles are not present to influence sexual orientation judgments. Based on previous studies, we expected a positive correlation among ratings of facial femininity and attributed homosexuality.

Finally, to test whether results of the previous studies can be generalized to a broader community, our raters consisted of non-students, unlike those in the majority of previous studies. This study was part of a broader project [28] aimed at romantic partner preferences of androphilic individuals and we therefore limited the set of raters to homosexual men and heterosexual women.****

## Materials and Methods

### 2.1 Ethics Statement

The research was approved by the IRB of the Charles University, Faculty of Science, and the study complies with the Declaration of Helsinki for Medical Research involving Human Subjects.

### 2.2 Target Participants

Facial photos and vocal recordings of 27 homosexual men (mean age 24.3 years; SD 4.71 years; range 18 – 35 years) and 34 heterosexual men (mean age 22.44 years; SD 2.81 years; range 19 – 30 years) were obtained for this study. The difference in age between homosexual and heterosexual men was not significant (t(40)  = 1.81, p = .078). All participants were of Caucasian appearance and except or two participants who were of Slovak descent (both of them were homosexual), they were Czech. The recordings of the Slovak participants were excluded from the voice-rating study. The participants completed a battery of questionnaires; for this study, only basic demographic information and sexual identification are relevant. Sexual identification was assessed using a 7-point Kinsey scale ranging from 0 (exclusively heterosexual) to 6 (exclusively homosexual). The distribution of the responses appeared to be dichotomous as none of the targets used points 2-4 to assess his sexual orientation. For further analyzes we, therefore, transformed the self-identification scale into two categories (heterosexuals, ratings of 0 and 1, and homosexuals, ratings of 5 and 6). Homosexual and heterosexual targets did not significantly differ in level and type of education but homosexual men reported significantly higher incomes than heterosexuals (t(40) =  5.134, p<.001).

The target sample of homosexual and heterosexual men was recruited through gay-oriented web pages, snowball, gay and homophile bars and clubs in Prague, and advertisements and leaflets distributed in various faculties of Charles University in Prague (for more details on sampling, see [28]. Data collection was performed by the first author within two months during the summer of 2006 in order to reduce possible seasonal effects (e.g. on skin color due to tanning).

Each participant received 300 CZK (approximately 17 US dollars) as compensation for their time, and all participants signed an informed consent form where they have been informed about the procedure, data treatment, were assured that data would be treated confidentially and solely for scientific purposes.

### 2.3 Facial Photographs

All data were collected under standardized conditions at the Laboratory of Human Ethology and facial photos and vocal recordings were taken after participants completed half of the questionnaires. All targets wore white T-shirts of appropriate size when photos were taken in order to standardize costume and reduce shadows in faces caused by colored clothes. Each participant used a black hair band to remove hair from the forehead. Further, they were asked to remove earrings, facial jewelry, and to adopt a neutral facial expression (neutral expression was defined as an expression when listening to a talk or walking on the streets). The portraits were taken using a Canon 350D camera with the focus Canon EF 50/1.8 II from a distance of 1.5 m. A light blue background Colorama was used to optimize white balance and subsequent digital adjustments. To eliminate possible influences of hair style, only faces without scalps were used for the ratings. The scalps were covered using Photoshop 7.0 software and faces were placed on a black background.

### 2.4 Vocal Stimuli and Acoustic Measurements

Vocal samples were recorded with a digital recorder, Olympus WS310M, with an external microphone, Sennheiser E845-S. Seated targets were asked to read aloud a standard paragraph of relatively emotionally neutral text with the microphone placed 10 to 15 cm from their mouth. The text (translated into Czech), described various historical concepts of a rainbow and was used in previous research on vocal attractiveness [8]. Each participant was familiarized with the text before the recordings were taken. To avoid potential effects of stress or fatigue, we extracted an intermediate section (approximately 20 s out of 90 to 120 s) of the recording, similar to the previous research [29].

SoundForge 8.0 software was used for the extraction and the volume standardization. All 59 voice samples were analyzed by Praat software (www.praat.org) for mean fundamental frequency (F0) that ranged from 86.4 to 191.8 Hz. Fundamental frequency was measured using Praat's autocorrelation algorithm with parameters set to a pitch floor of 75 Hz and a pitch ceiling of 300 Hz, with all other values set to default. Fundamental frequencies were averaged across sounds for each speaker.

### 2.5 Raters

Thirty eight women (mean age 23.2 years; SD = 5.3 years; range 18 – 39 years) and 39 non-heterosexual males (mean age 29.3 years; SD = 6.75 years; range 19 – 48 years) of Czech or Slovak origin and various socio-economic backgrounds took part in the rating session. Women were recruited in various public places in Prague (mostly open-air cafés), where it was possible to use the laptop. Male raters were recruited in gay bars, by using the snowball method and a leaflet distributed in gay internet sites, so that both individuals visiting gay bars and those who did not visit such places were recruited. The rating in gay bars occurred in afternoon hours and only individuals who had not been consuming alcohol were recruited.

Before the rating session, all raters completed a short digitalized questionnaire assessing their sexual orientation on a 7-point Kinsey scale (0 =  exclusively heterosexual; 6  =  exclusively homosexual) and basic demographic data. Out of 37 females who reported their sexual orientation, 22 rated themselves as exclusively heterosexual (number 0 on Kinsey scale), 14 as predominantly heterosexual (1 or 2 on Kinsey scale), and 1 rated herself as bisexual (3 on Kinsey scale). Seven of the female raters indicated that they had had at least one female sexual partner during their lifetime. Two male raters did not report their sexual orientation; 26 rated themselves as exclusively homosexual (6 on Kinsey scale), 10 as predominantly homosexual (5 or 4 on Kinsey scale), and 1 as bisexual (3 on Kinsey scale). One respondent stated he had a stable female sexual partner at the time of the study. Exclusion of the ratings given by the two bisexual individuals did not substantially affect the reported results. None of the raters were given monetary compensation for their participation.

### 2.6 Rating Procedure

Each rater first judged vocal recordings, and then the whole set of photographs. For the rating session, out of the whole dataset we randomly selected vocal recordings of 15 homosexual and 15 heterosexual men. The rest of the recordings were not used for the ratings because of time constraints for raters. To avoid carry-over effects, each rater assessed only one parameter. The stimuli were assessed for masculinity-femininity and sexual orientation on 7-point scale (1  =  masculine/heterosexual, 7  =  feminine/homosexual). In particular, masculinity-femininity was rated by 19 female and 20 homosexual male raters, and sexual orientation by 20 female and 19 homosexual male raters. Image ratings were carried out on a 17-inch laptop screen with 1280 x 800 pixel resolution using ImageRater 1.3 software specifically developed for these purposes. Vocal recordings were rated using headphones, Koss KSC/75.

### 2.7 Statistical Analyses

Cronbach’s alphas were used to assess inter-rater reliability. For male ratings of masculinity-femininity and sexual orientation of facial stimuli, the respective alpha values were.869, and.735, respectively, and for female ratings.750, and.676, respectively. Cronbach’s alpha for male ratings of masculinity-femininity and sexual orientation of vocal stimuli was.965, and.933, respectively, and for female ratings.861, and.916, respectively. Because Shapiro-Wilk's test showed deviations from a normal distribution in several data sets, we used non-parametric statistics if possible.

Female and homosexual male ratings of vocal (Kendall's tau  = .672, N = 30, p<.001) and facial masculinity-femininity (Kendall's tau  = .565, N = 61, p<.001), and vocal sexual orientation (Kendall's tau  = .616, N = 30, p<.001) showed significant positive correlations. However, the correlation of female and homosexual male ratings of the facial sexual orientation (Kendall's tau  = .164, N = 61, p = .065) showed only a non-significant trend. Since ratings of sexual orientation are central to this study, we further analyzed female and male ratings separately.

In order to test for predicted differences between homosexual and heterosexual targets in perceived masculinity-femininity and sexual orientation, we ran Wilcoxon Signed rank Test with mean ratings for each target as the unit of the analysis. All data analyses were performed using SPSS 20.0. The data are available upon request from the first author.

## Results

### 3.1 Attributions of sexual orientation

As shown in Table 1, vocal stimuli of homosexual and heterosexual targets significantly differed in their sexual orientation attributions as rated by both heterosexual female raters and homosexual male raters. Homosexual targets were judged higher on homosexuality than heterosexual targets.

**Table 1:** Differences in sexual orientation (SO) and masculinity-femininity (MF) attributions between heterosexual and homosexual targets

Judged parameter	Group of raters (N)	Mean attributions for heterosexual targets (SD)	M attributions for homosexual targets (SD)	Related-samples Wilcoxon Signed rank Test		Cohen's *d*
				Test statistic	p-value	
Vocal SO	Heterosexual women (20)	3.14 (.56)	3.68 (.61)	3.585	.000	.92
	Homosexual men (19)	3.51 (.52)	3.89 (.72)	3.139	.002	.61
Facial SO	Heterosexual women (20)	3.31 (.86)	3.05 (.74)	–2.072	.038	.32
	Homosexual men (19)	3.45 (.57)	3.78 (.57)	2.575	.010	.58
Vocal MF	Heterosexual women (19)	3.51 (.89)	3.46 (.84)	–.174	.862	.06
	Homosexual men (20)	3.62 (.45)	3.85 (.71)	1.531	.126	.39
Facial MF	Heterosexual women (20)	3.51 (.70)	3.29 (.77)	–2.294	.022	.30
	Homosexual men (20)	3.70 (.82)	3.40 (.75)	–3.061	.002	.38

Note. Ratings of masculinity-femininity and sexual orientation were performed using 7-point scale from 1 (masculine/heterosexual) to 7 (feminine/homosexual). Lower mean attributions notify higher scoring on masculinity or heterosexuality.

Based on facial stimuli, homosexual male raters judged male faces in accordance with their self-assessed sexual orientation, whereas female raters unexpectedly judged heterosexual targets significantly higher on homosexuality than they judged homosexual targets (see Table 1).

### 3.2 Perceived masculinity-femininity

As shown in Table 1, there were no significant differences in perceived vocal masculinity-femininity between heterosexual and homosexual target men as rated by both groups of raters. On the other hand, both groups of raters judged the faces of homosexual target men as significantly more masculine than the faces of heterosexual target men.

### 3.3 Correlations between judgments of masculinity-femininity and sexual orientation

We found significant positive correlations between the ratings of masculinity-femininity and sexual orientation based on vocal recordings as rated by both female and male raters (see Table 2). Similarly, we found significant positive correlations between the ratings of masculinity-femininity and sexual orientation based on facial photos as rated by both female and male raters. Finally, masculinity-femininity judged from faces did not significantly correlate with masculinity-femininity judged from voices neither in female (Kendall's tau  =  –.061, N = 30, p = .642) nor in male raters (Kendall's tau  = –.135, N = 30, p = .300). Similarly, attributions of sexual orientation from faces did not significantly correlate with attributions of sexual orientation from voices (female raters: Kendall's tau  = .072, N = 30, p = .580; male raters: Kendall's tau  = .063, N = 30, p<.629).

**Table 2:** Kendall’s Tau correlations between ratings of masculinity-femininity (MF) and sexual orientation (SO) based on ratings from vocal recordings (N = 30) and facial images (N = 61).

	Vocal MF	Vocal SO	Facial MF	Facial SO
Vocal MF		**.650** **	.111	.065
Vocal SO	**.574** **		.049	.100
Facial MF	.055	.021		**.309** **
Facial SO	.030	.091	**.354** **	

denotes p<.01.

Ratings by homosexual men are presented above the diagonal, and ratings by heterosexual women below the diagonal.

### 3.4 Effect of voice pitch on self-reported and attributed sexual orientation and masculinity-femininity based on vocal recordings

The mean fundamental frequency of the whole sample (N = 59) was 117 kHz (SD = 18.44), and there was no significant difference between homosexual and heterosexual targets in mean voice pitch (W = –.046, p = .963). Nonparametric correlations revealed a significant positive association between voice pitch and perceived masculinity-femininity as rated by both female (Kendall's tau  = .456, N = 30, p<.001) and male raters (Kendall's tau  = .386, N = 30, p = .003), and between voice pitch and male ratings of sexual orientation (Kendall's tau  = .328, N = 30, p = .011), but not by female raters (Kendall's tau  = .173, N = 30, p = .181).

## Discussion

This study builds upon previous research that showed perceived sexual orientation, based on voice stimuli, correlated significantly with self-reported sexual orientation [4]–[6], [24]. The current findings show that attributions of sexual orientation are congruent with self-reports and that they appear also in the Czech language, and are thus not limited to English. Previous studies have indicated that judgments of sexual orientation are linked to perceived gender-typical traits, and indeed, we found a positive correlation between perception of vocal femininity and attributed homosexuality. Thus, in agreement with the previous studies [18], [20], both women and homosexual men judged feminine-sounding voices as being homosexual and vice versa. Interestingly, judgments of sexual orientation were consistent with self-reported sexual orientation, even though homosexual and heterosexual targets did not significantly differ in attributed vocal masculinity-femininity. Our findings thus support the notion that even though correlated, perceived masculinity-femininity and sexual orientation are not perfectly linked [20]. Moreover, homosexual and heterosexual targets did not differ in voice pitch, but lower-pitched voices were perceived as more masculine and more heterosexual compared to higher-pitched voices. This finding is in agreement with previous studies that showed speakers judged as less masculine have higher formant frequencies, although not fundamental frequency [30], [31]. Thus, although low/high voice pitch strongly affects perception of vocal masculinity-femininity, there are apparently other vocal traits perceived as feminine that affect sexual orientation judgments.

Our study supports the previous findings on accuracy of attributions of sexual orientation based on facial images, but only in homosexual male raters, not in heterosexual female raters. Other results showed that female raters incorrectly attributed higher levels of homosexuality to heterosexual men, although the effect size of this result was relatively small. Similarly, one study showed that male homosexual raters perform better in detecting the sexual orientation of unknown individuals than heterosexual men [2], although several other studies did not find any sex difference in sexual orientation attribution [6], [14]. On the other hand, according to Shelp [2], homosexual men might develop an 'adaptive gaydar', since they have a higher experience with the minority and perhaps also higher motivation to recognize each other. Accordingly, a recent study has shown that familiarity with homosexual individuals increases accuracy in sexual orientation attribution based on facial images [32]. In a similar tone, we might argue that heterosexual women in our study were not sufficiently familiar with the homosexual community, and that they weren't motivated enough to pick up subtle facial cues which might be relevant for sexual orientation assessments. Although we did not specifically recruit individuals for familiarity with homosexual individuals, we may assume that in general homosexual men are more familiar with other homosexuals than heterosexual women. Correspondingly, ratings of individuals supposedly familiar with homosexual men showed higher agreement with self-reported sexual orientation of the rated men, whereas ratings of individuals supposedly less familiar with homosexual individuals showed an opposite pattern.

Another result showed that, contrary to our expectations, both groups of raters judged faces of homosexual targets to be more masculine than those of heterosexual men, although effect sizes of these results were rather small. This might explain why female raters judged homosexual targets higher on heterosexuality than heterosexual targets. Perceived masculinity-femininity thus appear to be stereotypically associated with sexual orientation judgments, nevertheless, if homosexual men do not show stereotypically feminine traits, then judgments of sexual orientation may not correspond to the self-reported sexual orientation, as was presumably the case in our study. Thus, women in our sample might have relied rather on the stereotypic association of perceived male femininity and homosexual orientation. In comparison, homosexual men, who are expected to be more familiar with facial appearance of other homosexual men, did not entirely rely on the stereotype of a feminine gay man. This interpretation is consistent with recent studies showing that rather feminine heterosexuals are reliably rated as homosexuals, whereas gender-typical homosexuals are rated as heterosexuals [10], [33]. Furthermore, in another recent investigation, raters were most accurate in their ratings of sexual orientation from facial pictures of the gender-stereotyped groups of individuals, and least accurate when they assessed gender counter-stereotypic groups (in this case, the gender-stereotypic groups were defined by their race) [19]. These results show that sexual orientation judgments based on stereotyped gender-specific traits can lead to frequent misjudgments, in particular because not all homosexual individuals show gender nonconforming traits (for review on the relationship between childhood gender nonconformity and sexual orientation, see e.g. Bailey and Zucker [16]). Perhaps, we might have coincidentally recruited rather gender-conforming homosexual men, or more feminine heterosexual men, which could have led to systematic misjudgments in female raters, although not in gay male raters. The homosexual participants in our study were mostly recruited via gay-oriented web pages and snowball technique. Although obtaining a representative sample of sexual minorities is technically difficult, if impossible, one should nevertheless consider putative sampling bias in the reported results. Homosexual men frequenting gay oriented web pages and those willing to participate in a study aimed at sexual orientation may differ from homosexual men recruited e.g. via lonely-heart advertisements. Thus, in some groups of homosexual men, masculine traits might be more pronounced than feminine traits. Future studies on 'gaydar' should therefore take into account various subgroups of homosexual minority, such as feminine 'twinks' or masculine 'bears' [34].

We thus suggest that studies on sexual orientation judgments should control for self-rated and other-rated masculinity-femininity of the targets. People's judgments are frequently based on gender-related stereotypes, and the positive results might be limited to gender-stereotypic homosexual men (i.e. feminine homosexual men), who might be perceived differently than gender-stereotypic heterosexual men (i.e. masculine heterosexual men). This might also be the reason why the efficiency of the 'gaydar' documented in previous studies is rather low, i.e., there might be confusing subgroups of homosexual individuals which do not follow the widespread stereotypes, and in some populations, these groups might be more prevalent than in others. In other words, although on average, homosexual men are more feminine than heterosexuals, not all homosexual men are feminine, and not all feminine men are homosexuals [35]. It is thus possible that at least two very distinct subgroups of homosexual men based on their gender nonconformity (i.e. feminine and masculine homosexual men) are studied as a single phenomenon.

Interestingly, in our previous study [14], we have shown that Czech individuals were able to infer male sexual orientation based on short videos, and this functioned to some degree cross-culturally. More specifically, both American and Czech raters were able to infer sexual orientation of both Czech and US men, although the correlations with actual sexual orientation within each population were stronger than across cultures. Furthermore, in another study, which was partially based on the same target stimuli as this study, we found no significant association between sexual orientation ratings of facial photos and self-reported male sexual orientation [36]. In this study, heterosexual male and female raters were recruited among students. However, the null findings are in line with the results from community-based sample of the heterosexual females in the present study. Thus, we might speculate that when judging male sexual orientation, Czech heterosexual individuals use more efficiently dynamic cues, either based on behavioral cues or voice, rather than more static facial features. Nevertheless, more research based on samples from diverse populations is needed to establish generalizability of the current research.

Differences between our results and those of previous studies might also be attributed to the fact that raters in our study were recruited among a community sample, i.e. mostly non-student population. Our aim was to test whether previous findings based on student samples are generalizable to a broader community. Although we acknowledge that the current sample is not highly representative, the results showed that sexual orientation judgments based on voices are accurate above chance-level when non-student raters are employed. In contrast, we did not find similar effects with facial stimuli. In the current study, we used highly standardized portraits with a neutral facial expression and with covered hair to minimize social cues, whereas some previous studies employed non-standardized stimuli taken from internet personal ads (e.g., Facebook [11] or images edited from videos [7]). Non-standardized stimuli may contain additional cues such as hair-style and facial expression, which could serve as cues to sexual orientation. Both methods have advantages and disadvantages: standardized pictures could conceal any obvious cues to sexual orientation.

Although some of the differences between our study and previous studies discussed at length above might have affected the results of the present study, it should be stressed that, in fact, the majority of our results correspond to those reported previously. First, we have shown that sexual orientation is effectively attributed from vocal stimuli, thus extending the findings on vocal 'gaydar' into the non-Germanic language families. Secondly, attributed vocal and facial femininity was associated with attributed homosexuality. Finally, our study has also supported previous work on accuracy of facial attributions of sexual orientation, although this applied only to homosexual male raters. The two other findings are in conflict with previous studies. More specifically, women did not accurately judge male sexual orientation, and faces of self-identified homosexual men were rated as more masculine than heterosexuals. As we have noted earlier, only by conducting similar studies in different populations, and in particular under different methodological conditions, we will be able to draw more general patterns of the studied phenomena [26]. Thus, we stress that more cross-cultural studies using a variety of paradigms, and particularly sampling directly aimed at different gay subcultures, might shed more light on the psychological processes related to 'gaydar'.
